# Non-invasive biomarkers for early diagnosis of pancreatic cancer risk: metabolite genomewide association study based on the KCPS-II cohort

**DOI:** 10.1186/s12967-023-04670-x

**Published:** 2023-12-04

**Authors:** Youngmin Han, Keum Ji Jung, Unchong Kim, Chan Il Jeon, Kwangbae Lee, Sun Ha Jee

**Affiliations:** 1 Institute for Health Promotion, Graduate School of Public Health, Yonsei University, 50 Yonsei-Ro, Seodaemun-Gu, Seoul, 03722 Republic of Korea; 2 Korea Medical Institute, Seoul, Republic of Korea

**Keywords:** Pancreatic cancer, Predictive biomarker, Genetic variants, LC/MS metabolomics, Metabolite genomewide association study

## Abstract

**Background:**

Pancreatic cancer is a lethal disease with a high mortality rate. The difficulty of early diagnosis is one of its primary causes. Therefore, we aimed to discover non-invasive biomarkers that facilitate the early diagnosis of pancreatic cancer risk.

**Methods:**

The study subjects were randomly selected from the Korean Cancer Prevention Study-II and matched by age, sex, and blood collection point [pancreatic cancer incidence (*n* = 128) *vs.* control (*n* = 256)]. The baseline serum samples were analyzed by non-targeted metabolomics, and XGBoost was used to select significant metabolites related to pancreatic cancer incidence. Genomewide association study for the selected metabolites discovered valuable single nucleotide polymorphisms (SNPs). Moderation and mediation analysis were conducted to explore the variables related to pancreatic cancer risk.

**Results:**

Eleven discriminant metabolites were selected by applying a cut-off of 4.0 in XGBoost. Five SNP presented significance in metabolite-GWAS (*p* ≤ 5 × 10^–6^) and logistic regression analysis. Among them, the pair metabolite of rs2370981, rs55870181, and rs72805402 displayed a different network pattern with clinical/biochemical indicators on comparison with allelic carrier and non-carrier. In addition, we demonstrated the indirect effect of rs59519100 on pancreatic cancer risk mediated by γ-glutamyl tyrosine, which affects the smoking status. The predictive ability for pancreatic cancer on the model using five SNPs and four pair metabolites with the conventional risk factors was the highest (AUC: 0.738 [0.661–0.815]).

**Conclusions:**

Signatures involving metabolites and SNPs discovered in the present research may be closely associated with the pathogenesis of pancreatic cancer and for use as predictive biomarkers allowing early pancreatic cancer diagnosis and therapy.

**Supplementary Information:**

The online version contains supplementary material available at 10.1186/s12967-023-04670-x.

## Introduction

The pancreas is an organ responsible for producing digestive juices and regulating the blood glucose levels. Pancreatic cancer is very lethal considering that early diagnosis is challenging and the chances of metastasis to the other organs are very high [1]. Pancreatic cancer accounts for approximately 3% of all cancers in the United States, and it is more common in men than in women [2]. According to the National Statistical Office of Korea, 6931 people (3600 men and 3331 women) died from pancreatic cancer, accounting for 8.4% of all cancer cases in 2021 [3].

The cause of pancreatic cancer is unclear, but smoking, being overweight, diabetes, and a relevant family history act as risk factors for pancreatic cancer. Smoking is a crucial risk factor for chronic pancreatic cancer [4]. In a study involving 2009 pancreatic cancer cases and 1532 control groups from the International Pancreatic Cancer Cohort, smokers showed a 1.72-fold higher risk of pancreatic cancer than the non-smokers. In addition, as per a report, the more the numbers of cigarettes smoked, the higher the risk of pancreatic cancer [5].

Recently, several studies were conducted on pancreatic cancer. Currently, the most widely used single tumor marker for pancreatic cancer is carbohydrate antigen (CA) 19–9, as noted in 80% of all pancreatic cancer patients. However, as its specificity is low for screening tests, it is usually used to determine the stage and prognosis of pancreatic cancer or to monitor its recurrence [6, 7]. In addition, Hwang et al. [8] suggested that the miR-21 expression is closely related to anticancer drug resistance; this aspect can be applied to predict anticancer drug resistance and the clinical outcomes for Korean pancreatic cancer patients. However, there are no biomarkers for the early diagnosis or early detection of pancreatic cancer risk yet.

Multi-omics is a method of comprehensively analyzing the data generated at various molecular levels, such as genome, transcriptome, proteome, and metabolome; it has been applied in multiple fields for disease research [9, 10]. This approach can provide systemic clues to understand the underlying metabolic changes occurring through the disease duration. Indeed, proteomics on genetically engineered mouse models with early and advanced stages of pancreatic cancer identified candidate proteome markers applicable to early detection [11]. Moreover, for ovarian cancer that was mainly diagnosed in the late stage, multi-omics technology has been widely used to discover several valuable biomarkers for the early diagnosis [12].

This study aims to discover non-invasive biomarkers for predicting pancreatic cancer risk through multi-omics technology. Genotyping and non-targeted screened metabolite data in the Korean subjects from the Korean Cancer Prevention Study (KCPS)-II were integratively analyzed through diverse statistical analyses. We expected that, our findings, including genomic and metabolomic biomarkers, can serve as the basis for research on pancreatic cancer pathogeneses.

## Materials and methods

### Study population

The study subjects were selected from the KCPS-II cohort. Briefly, the KCPS-II subjects were recruited through 18 health promotion centers across South Korea from April 2004. After their enrollment, hospital admission records, death registries, and National Cancer Center registry data were collected during the follow-up period. Written informed consent for cohort registration and secondary research was obtained from all cohort subjects, and their blood samples were collected.

For the current research, subjects aged 25–71 years were randomly selected from the KCPS-II. We comprised two groups by matching in a 1:2 ratio by age, sex, and the blood collection point [pancreatic cancer incidence group (*n* = 128) *vs.* control (*n* = 256)]. The subjects who were cancer-free at the time of enrollment, but later developed pancreatic cancer during the follow-up period were assigned to the case group.

All procedures in the current research involving human participants were performed in accordance with the ethical standards of the Institutional Review Board at the Yonsei University Health System under the Helsinki Declaration [IRB Number: 4-2022-1136].

### Smoking history

Each participant answered a self-administered questionnaire concerning their smoking habits (never-smoker = 0, ex-smoker = 1, or a current smoker = 2). The smoking amount of current smokers was also investigated, but due to several missing values, this data was not used in this study.

## Metabolome analysis

### Non-targeted metabolomics

#### UHPLC-MS/MS analysis

The prepared serum samples were precipitated with cold acetonitrile (Wako Pure Chemical Industries, Osaka, Japan) (1:3, *v*/*v*) and centrifuged for 15 min (13,000 rpm, 4 ℃). The supernatant was then separated and dried in a vacuum concentrator (HyperVAC-MAX, Hanil Scientific Inc., Gimpo, Korea) without heating. Next, 200 μL of 10% methanol (J.T. Baker^®^ Chemicals; Avantor Performance Materials, Inc., Radnor, PA, USA) was added for reconstitution and filtrated through a 0.45-μm polyvinylidene difluoride syringe filter. L-Leucine-1-^13^C (Sigma-Aldrich, Saint Louis, MO, USA) was used as an internal standard (ISTD). The quality control (QC) sample was prepared following the exact step by combining all the serum samples.

The serum samples were injected into the Acquity UPLC-BEH-C18 column (Waters, Milford, MA, USA) connected to the Thermo UHPLC system (Ultimate 3000 BioRS; Dionex, Thermo Fisher Scientific, Bremen, Germany). The column temperature was maintained at 50 ℃. Two mobile phases [**A**, composed of 0.1% formic acid in LC–MS grade water (Thermo Fisher Scientific, Fair Lawn, NJ, USA); **B**, composed of 0.1% formic acid in LC–MS-grade methanol (Thermo Fisher Scientific, Fair Lawn, NJ, USA)] made gradient during 17 min for separating the compounds in the samples. Q Exactive Plus Orbitrap (Thermo Fisher Scientific, Waltham, MA, USA) was combined with the UHPLC system for data detection. On MS, positive electrospray ionization mode (ESI +) with 30 of collision energy, 3.5 kV of spray voltage, 60 (arbitrary units) of a flow rate of nitrogen sheath gas, and 20 (arbitrary units) of a flow rate of auxiliary gas was performed. Full scan-ddms^2^ mode with a scan range of 80–1000 mass-to-charge (*m/z*) was used to collect data.

The QC samples were measured for every 10th prepared serum sample and monitored for sensitivity and reproducibility. In addition, the intra-assay and inter-assay variations were assessed using replicated results of QC samples for a few days.

#### Identification of metabolites

Compound Discoverer 3.2 software (Thermo Fisher Scientific, San Jose, CA, USA) was used for processing the raw spectra. Alignment and normalization were performed QCs in the program. Features detected < 80% in all QC samples were discarded. Processed features were identified with reference to online databases ChemSpider (http://www.chemspider.com), LIPID MAPS (https://www.lipidmaps.org), mzCloud (https://www.mzcloud.org), and Kyoto Encyclopedia of Genes and Genomes (KEGG; https://www.genome.jp/kegg).

### Genotyping

DNA was genotyped using the KORV1.0–96 Array (Affymetrix, Santa Clara, CA, USA) provided by the K-CHIP consortium and Affymetrix Genomewide Human SNP Array 5.0 (Affymetrix Inc.). Markers with a high missing rate (> 5%), individuals with a high missing rate (> 5%), and SNPs with a minor allele frequency < 0.05 or in a significant deviation from the Hardy—Weinberg equilibrium (*p* < 1.0E − 6) were excluded for quality control.

### Statistical analysis

All statistical analyses were conducted by SPSS 26 (IBM Corp, Armonk, NY, USA), R 4.1.3, and Python 3.9.12. We performed Independent *t*-tests and Mann–Whitney U-tests to evaluate the differences in the clinical/biochemical variables between the two groups. The skewed variables were logarithmically transformed. For nominal variables, a Chi-square test was applied. The data are expressed as the mean ± SE, and two-tailed *p* < 0.05 were considered to indicate statistical significance.

For multivariate analyses, the normalized metabolite data were exported from Compound Discoverer 3.2. After Pareto-scaling and logarithmically transforming, the eXtreme Gradient Boosting (XGBoost) model was fitted using Python. The log-loss function was applied as the target in a binary variable (control; 0, case; 1). To optimize the model hyperparameters, we limited the maximum depth of the trees and eta while increasing the n_estimators so as to help prevent overfitting; the model using a too-small weak learner (n_estimators) with deep tree may contain noise, and reducing the eta diminishes the contribution of each tree to the model. As a result, the XGBoost model was fitted with the following parameters to achieve a high AUC in the test set: n_estimators, 50; learning rate, 0.15, alpha, 0.001; max depth, 2; min child weigh, 5; and et, 0.1.

Metabolite-GWAS was performed using PLINK 2.0. Next, logistic regression analysis was performed after adjusting for age and sex to evaluate the association between the revealed significant SNPs and pancreatic cancer. The predictive ability for pancreatic cancer using the biomarkers discovered in this study was assessed through regression analysis. Furthermore, we confirmed whether the smoking status is a significant moderator of the association between metabolites (independent variable) and pancreatic cancer incidence (dependent variable) by using *p-*values from a coefficient of the interaction term (metabolites* smoking status). In addition, we conducted a mediation analysis to demonstrate a metabolite as a significant mediator of the association between smoking status (independent variable) and pancreatic cancer incidence (dependent variable) using the R *mediate* function in the *mediation* package. Python and R codes used in the current research were provided in Additional file 2: Data S1.

A network model was created in the carrier and the non-carrier groups of effect alleles so as to visualize the relationships between clinical/biochemical indicators and paired metabolites of each SNP based on partial correlation. To reflect the difference in the quantitative abundance between the pancreatic cancer incidence and control groups, we calculated the z-score of each variable.

## Results

### Anthropometric and clinical/biochemical characteristics at the baseline

Excluding 35 subjects without genotyping data, 349 patients were included in the final analysis [pancreatic cancer incidence group (*n* = 113) *vs.* control (*n* = 236)]. The baseline characteristics of the total subjects are presented in Table 1. No significant differences were noted between the pancreatic cancer incidence and control groups. To summarize, the mean age was 52.4 years in the pancreatic cancer incidence group and 52.7 years in the control group (*p* from *t*-test = 0.991). The pancreatic cancer incidence group included 77.0% male and 23.0% female, while the control group included 73.7% male and 26.3% female, indicating no significant difference between the groups (*p* = 0.511). No statistical difference was noted in BMI, with the pancreatic cancer incidence and control groups showing respective mean values of 24.6 and 24.3 (*p* = 0.238). In addition, the two groups showed no significant difference in CA 19–9 (pancreatic cancer incidence group, 20.0 ± 2.48; control group, 8.37 ± 0.526; *p* = 0.346). The Chi-squared test confirmed the lack of any significant difference in the frequency of current smokers between the two groups (pancreatic cancer incidence group, 31.7%; control group, 30.3%; *p* = 0.116).

**Table 1:** Baseline clinical and biochemical characteristics of subjects

	Total (*n* = 349)		*p*
	Control (*n* = 236)	Pancreatic cancer incidence (*n* = 113)	
Age (year)	52.4 ± 0.588	52.7 ± 0.832	0.991
Male/female *n,* (%)	174 (73.7)/62 (26.3)	87 (77.0)/26 (23.0)	0.511
Current smoker *n,* (%)	70 (30.3)	33 (31.7)	0.116
Body mass index (kg/m^2^)^*†*^	24.3 ± 0.187	24.6 ± 0.256	0.238
Systolic blood pressure (mmHg)^*†*^	120.8 ± 0.940	121.2 ± 1.24	0.929
Diastolic blood pressure (mmHg)^*∮*^	75.0 ± 0.701	76.7 ± 0.833	0.571
Glucose (mg/dL)^*†*^	95.6 ± 1.37	101.4 ± 2.90	0.439
White blood cell (10^3^/μL)^*†*^	5.99 ± 0.109	8.26 ± 2.14	0.536
Albumin (g/dL)^*†*^	4.53 ± 0.017	4.51 ± 0.027	0.199
Total cholesterol (mg/dL)^*∮*^	193.6 ± 2.28	192.0 ± 3.27	0.669
Triglyceride (mg/dL)^*∮*^	151.5 ± 6.79	141.1 ± 7.04	0.686
HDL-cholesterol (mg/dL)^*†*^	50.3 ± 0.717	49.4 ± 1.01	0.286
LDL-cholesterol (mg/dL)^*†*^	115.8 ± 2.08	117.0 ± 2.95	0.789
AST (IU/L)^*†*^	26.0 ± 1.48	24.9 ± 0.847	0.986
ALT (IU/L)^*†*^	27.4 ± 2.18	27.2 ± 1.50	0.353
GGT (IU/L)^*†*^	41.8 ± 3.44	41.8 ± 4.20	0.574
ALP (IU/L)^*†*^	121.5 ± 4.49	131.4 ± 6.84	0.223
Bilirubin (mg/dL)^*†*^	0.881 ± 0.023	0.894 ± 0.040	0.710
Uric acid (mg/dL)	5.56 ± 0.092	5.39 ± 0.123	0.282
Blood urea nitrogen (mg/dL)^*†*^	14.6 ± 0.237	14.6 ± 0.337	0.722
Creatinine (mg/dL)^*†*^	1.00 ± 0.013	0.998 ± 0.017	0.164
CA 19–9 (U/mL)^*†*^	8.37 ± 0.526	20.0 ± 2.48	0.346

Mean ± standard error (SE). Comparisons were conducted between the two groups (control *vs.* pancreatic) cancer incidence). Continuous variables were tested by an independent t-test, and variables marked with *∮* were tested by logarithmic transformation. Continuous variables with a nonnormal distribution, even after logarithmic transformation, were tested by a Mann–Whitney U test, and *p*-values are marked with *†*. Smoking status was tested by a Chi-squared test

*AST* aspartate aminotransferase, *ALT* alanine aminotransferase, *GGT* γ-glutamyltransferase, *ALP* alkaline phosphatase, *HDL* high-density lipoprotein, *LDL* low-density lipoprotein

### Discriminant metabolites between the pancreatic cancer incidence and control groups

Among the 3165 detected features from MS, 173 metabolites were identified. A heatmap comparing the abundance of identified metabolites between the pancreatic cancer incidence and control groups is shown in Additional file 1: Figure S1.

Before establishing the XGBoost model, a random seed 6:4 was applied to divide the training and the test sets (Additional file 2: Data S2). In the training set, 68 individuals from the pancreatic cancer incidence group and 141 from the control group were included. There was no significant difference in the age and sex distribution between these two groups. The proportion of current smokers in the pancreatic cancer incidence group was 30.9%, which showed a statistical difference from the control group of 30.5% (*p* = 0.018). In the test set, 45 individuals were from the pancreatic cancer incidence group, while 95 were from the control group. There were no significant differences in terms of age, gender, or smoking status between these two groups. 

We fitted XGBoost on the training dataset (*n* = 209) and calculated the feature importance for identifying the effect of metabolites on the fitted model. As a result, 11 metabolites that considerably differed between the groups were selected (feature importance ≥ 4.0), as summarized in Table 2. The levels of serum eicosa-11,14,17-trienoic acid, kynurenic acid, γ-glutamyl tyrosine, lysoPE(18:0/0:0), trans-3'-hydroxy cotinine, and L-leucine were found to be elevated in the pancreatic cancer incidence group. In contrast, the pancreatic cancer incidence group had lower N(6)-methyllysine, palmitic amide, adipic acid, 9-decenoylcarnitine, and 5α-pregnane-3,20-dione levels than the control group.

**Table 2:** Identification of meaningful metabolites using XGBoost

Putative identification	HMBD ID	*m/z*	Formula	Feature importance	Pancreatic cancer incidence/control
Eicosa-11,14,17-trienoic acid	HMDB0244373	306.2560	C_20_H_34_O_2_	6.0	1.826
Kynurenic acid	HMDB0000715	189.0429	C_10_H_7_NO_3_	6.0	1.069
γ-Glutamyl tyrosine	HMDB0011741	310.1166	C_14_H_18_N_2_O_6_	5.0	1.230
N(6)-Methyllysine	HMDB0002038	160.1214	C_7_H_16_N_2_O_2_	5.0	0.875
LysoPE(18:0/0:0)	HMDB0011130	481.3170	C_23_H_48_NO_7_P	5.0	1.040
Trans-3'-hydroxy cotinine	HMDB0304504	192.0901	C_10_H_12_N_2_O_2_	4.0	1.130
Palmitic amide	HMDB0012273	255.2563	C_16_H_33_NO	4.0	0.915
L-Leucine	HMDB0000687	131.0949	C_6_H_13_O_2_	4.0	1.144
Adipic acid	HMDB0000448	146.0581	C_6_H_10_O_4_	4.0	0.795
9-Decenoylcarnitine	HMDB0013205	313.2254	C_17_H_31_NO_4_	4.0	0.794
5α-Pregnane-3,20-dione	HMDB0003759	316.2398	C_21_H_32_O_2_	4.0	0.845

Feature Importance values ​​ > 4.0 are listed in Table 2. Feature Importance value was obtained from the XGBoost model of the training set (*n* = 209) [accuracy, 0.952; precision, 0.985; AUC 0.998], selecting discriminant metabolites related to pancreatic cancer incidence. The pancreatic cancer incidence/Control value was calculated using the relative abundance of each metabolite

The performance values of the XGBoost model on the training and test sets are shown in Additional file 2: Data S2. The training set had an accuracy of 0.952, precision of 0.983, recall of 0.868, and AUC of 0.998. In the case of the test set, an accuracy of 0.671, precision of 0.471, recall of 0.178, and AUC of 0.640 were recorded.

### Metabolite-genomewide association analysis

Using 11 selected metabolites, we conducted a metabolite-GWAS. We generated a Manhattan plot to identify significant SNPs and performed linkage disequilibrium clumping with a threshold of *p* ≤ 5 × 10^–6^ to mitigate the tendency for correlation between genetic variants located nearby. Logistic regression analysis was performed to demonstrate their association with the incidence of pancreatic cancer (Table 3). Particularly, the G allele of rs2370981 mapped to *NRXN3*, strongly related to eicosa-11,14,17-trienoic acid, was identified as a protective allele for pancreatic cancer [OR = 0.371, *p* = 0.043]. Other four notable SNPs (i.e., rs59519100, rs11164375, rs72805402, and rs55870181) were all associated with a higher risk of pancreatic cancer; rs59519100 showed a significant association with γ-glutamyl tyrosine, rs11164375 with lysoPE (18:0/0:0), rs72805402 (mapped to *ZNF503)* and rs55870181 with L-leucine; Manhattan plots for these are presented in Additional file 1: Figure S2.

**Table 3:** Genome-wide association analysis of pancreatic cancer-related metabolites

Metabolites	SNP	Position	EA	EAF	Mapped Gene	OR [CI]	*p*
HMDB0244373 Eicosa-11,14,17-trienoic acid	rs6731366	chr2:132891267 (GRCh38.p14)	A	0.053	*NCKAP5*	1.740 [0.833–3.633]	0.141
	rs11860247	chr16:16071432 (GRCh38.p14)	A	0.086	*ABCC1*	1.016 [0.534–1.934]	0.960
	rs201237448	chr3:157725970 (GRCh38.p14)	A	0.053		0.869 [0.388–1.944]	0.732
	rs4541064	chr16:85230276 (GRCh38.p14)	C	0.487	*GSE1*	1.037 [0.615–1.748]	0.892
	rs114089627	chr3:3658708 (GRCh38.p14)	A	0.016		1.106 [0.503–2.432]	0.802
	rs138810234	chr4:161529556 (GRCh38.p14)	C	0.036	*FSTL5*	1.430 [0.834–2.450]	0.193
	rs77806269	chr3:12881670 (GRCh38.p14)	T	0.023	*LINC02022, LOC105376956*	0.749 [0.363–1.548]	0.436
	**rs2370981**	chr14:79402892 (GRCh38.p14)	G	0.017	*NRXN3*	0.371 [0.142–0.968]	**0.043**
	rs117753991	chr16:77579192 (GRCh38.p14)	G	0.024		0.951 [0.518–1.748]	0.873
	rs201592606	chr4:139691697 (GRCh38.p14)	G	0.050	*MGST2*	1.089 [0.683–1.738]	0.720
HMDB0000715 Kynurenic acid	rs73448444	chr13:28731007 (GRCh38.p14)	G	0.138		1.075 [0.614–1.883]	0.799
	rs200475458	chr17:16403690–703 (GRCh38.p14)	C	0.110		0.833 [0.463–1.498]	0.541
	rs187490	chr5:35045022 (GRCh38.p14)	G	0.311	*AGXT2*	1.256 [0.787–2.005]	0.339
	rs604140	chr2:67422625 (GRCh38.p14)	C	0.050		1.734 [0.834–3.602]	0.140
	rs78053646	chr12:231126 (GRCh38.p14)	T	0.166	*SLC6A13, PARM1*	1.233 [0.746–2.039]	0.414
	rs890289	chr4:75,047,822 (GRCh38.p14)	A	0.054	*LOC107986289*	0.998 [0.481–2.070]	0.995
	rs9908634	chr17:79431371 (GRCh38.p14)	T	0.062	*RBFOX3*	1.224 [0.603–2.485]	0.576
	rs12909308	chr15:61777037 (GRCh38.p14)	T	0.284		1.223 [0.770–1.942]	0.393
	rs10407389	chr19:36484416 (GRCh38.p14)	G	0.103	*ZNF566*	0.996 [0.559–1.775]	0.990
	rs72747726	chr15:69,899,377 (GRCh38.p14)	G	0.238		1.093 [0.680–1.757]	0.712
HMDB0011741 γ-Glutamyl tyrosine	**rs59519100**	chr20:33868761 (GRCh38.p14)	T	0.166		1.701 [1.046–2.765]	**0.032**
	rs193488	chr5:136703440 (GRCh38.p14)	G	0.053		1.920 [0.948–3.890]	0.070
	rs202074299	chr13:90,574,203 (GRCh38.p14)	T	0.165		1.126 [0.689–1.839]	0.637
HMDB0002038 N(6)-Methyllysine	rs200559669	chr6:123584482 (GRCh38.p14)	C	0.481	*TRDN*	0.685 [0.415–1.131]	0.139
	rs918171	chr19:3336541 (GRCh38.p14)	C	0.370		0.947 [0.599–1.499]	0.817
	rs2374205	chr4:113984965 (GRCh38.p14)	G	0.143	*LOC124900762*	0.893 [0.523–1.524]	0.677
	rs116931887	chr6:143808387 (GRCh38.p14)	C	0.384	*PHACTR2*	0.971 [0.612–1.241]	0.900
HMDB0011130 LysoPE(18:0/0:0)	rs11083238	chr18:27943526 (GRCh38.p14)	T	0.135	*CDH2*	1.299 [0.744–2.267]	0.358
	rs6731366	chr2:132891267 (GRCh38.p14)	A	0.053	*NCKAP5*	1.329 [0.615–2.874]	0.469
	rs12059514	chr1:102370790 (GRCh38.p14)	C	0.181		0.987 [0.571–1.706]	0.964
	rs28565987	chr15:88048299 (GRCh38.p14)	A	0.097	*NTRK3*	0.829 [0.432–1.590]	0.573
	rs2505110	chr10:30181971 (GRCh38.p14)	G	0.248		0.843 [0.514–1.384]	0.500
	rs62525721	chr8:129066916 (GRCh38.p14)	T	0.052		0.986 [0.430–2.259]	0.973
	rs8052560	chr16:88710834 (GRCh38.p14)	C	0.082	*CTU2*	1.391 [0.734–2.636]	0.312
	rs117723718	chr4:40487925 (GRCh38.p14)	G	0.057	*RBM47*	1.050 [0.489–2.254]	0.901
	rs28705703	chr6:167502412 (GRCh38.p14)	G	0.066		1.650 [0.823–3.306]	0.158
	rs9829051	chr3:31491669 (GRCh38.p14)	G	0.122		1.149 [0.628–2.102]	0.652
	rs375927045	chr7:16220625 (GRCh38.p14)	C	0.208	*CRPPA*	0.624 [0.370–1.052]	0.077
	rs881433	chr18:44864508 (GRCh38.p14)	A	0.262	*SETBP1*	1.293 [0.799–2.092]	0.295
	**rs11164375**	chr1:102083600 (GRCh38.p14)	T	0.080		2.194 [1.095–4.394]	**0.027**
	rs117753153	chr3:24689035 (GRCh38.p14)	G	0.050		1.041 [0.446–2.427]	0.927
	rs1923773	chr13:53176219 (GRCh38.p14)	G	0.148		0.999 [0.576–1.732]	0.996
	rs141483946	chr11:13570434 (GRCh38.p14)	A	0.079		1.472 [0.751–2.884]	0.260
	rs9345335	chr6:93188629 (GRCh38.p14)	G	0.400		0.999 [0.605–1.651]	0.998
	rs78505433	chr15:49737092 (GRCh38.p14)	T	0.053		1.642 [0.761–3.547]	0.207
	rs57966757	chr18:5919654 (GRCh38.p14)	A	0.076	*LOC121725015*	0.938 [0.477–1.844]	0.853
	rs4727289	chr7:93397016 (GRCh38.p14)	G	0.120		0.743 [0.407–1.356]	0.333
	rs55721115	chr14:34277292 (GRCh38.p14)	G	0.054		0.515 [0.212–1.250]	0.143
HMDB0304504 Trans-3′-hydroxy cotinine	rs74600139	chr5:44446171 (GRCh38.p14)	C	0.249		0.845 [0.531–1.344]	0.477
	rs8100204	chr19:19282905 (GRCh38.p14)	A	0.201	*SUGP1*	0.763 [0.472–1.234]	0.270
	rs148195640	chr20:59240551 (GRCh38.p14)	T	0.053	*ZNF831*	0.754 [0.435–1.306]	0.313
	rs4308248	chr3:134302128 (GRCh38.p14)	G	0.129		0.729 [0.327–1.621]	0.438
HMDB0012273 Palmitic amide	rs13043798	chr20:23283897 (GRCh38.p14)	A	0.269		0.709 [0.446–1.127]	0.146
	rs13132855	chr4:44399250 (GRCh38.p14)	A	0.085	*KCTD8*	1.038 [0.551–1.954]	0.908
	rs7949816	chr11:60278427 (GRCh38.p14)	A	0.130		0.778 [0.453–1.338]	0.364
	rs2724067	chr7:93831676 (GRCh38.p14)	A	0.246		0.877 [0.552–1.393]	0.579
	rs149210546	chr4:8824426 (GRCh38.p14)	C	0.391		1.458 [0.896–2.375]	0.129
	rs76582834	chr4:164383443 (GRCh38.p14)	C	0.076	*MARCHF1*	0.568 [0.280–1.152]	0.117
HMDB0000687 l-Leucine	rs76417681	chr2:61231241 (GRCh38.p14)	C	0.062	*USP34*	0.610 [0.271–1.371]	0.232
	rs17684350	chr10:18374682 (GRCh38.p14)	C	0.080	*CACNB2*	1.597 [0.814–3.133]	0.173
	rs55828915	chr1:207785790 (GRCh38.p14)	T	0.060	*CD46*	0.742 [0.335–1.642]	0.462
	rs57192942	chr10:127846701 (GRCh38.p14)	T	0.265		1.415 [0.863–2.320]	0.169
	rs72709073	chr9:69206346 (GRCh38.p14)	C	0.054	*TJP2*	0.749 [0.331–1.696]	0.488
	rs7182182	chr15:54,330,440 (GRCh38.p14)	A	0.064	*UNC13C*	0.848 [0.395–1.822]	0.673
	rs11525305	chr10:6632812 (GRCh38.p14)	A	0.073	*LINC02648*	1.365 [0.688–2.708]	0.373
	rs74724211	chr19:44209431 (GRCh38.p14)	G	0.097	*ZNF227*	0.847 [0.447–1.605]	0.610
	rs79500165	chr2:141763014 (GRCh38.p14)	T	0.056	*LRP1B, LOC107985779*	0.663 [0.271–1.626]	0.370
	**rs72805402**	chr10:75306714 (GRCh38.p14)	A	0.152	*ZNF503*	2.150 [1.258–3.674]	**0.005**
	rs687168	chr17:14441416 (GRCh38.p14)	C	0.172		1.281 [0.657–2.495]	0.467
	rs13388819	chr2:64599202 (GRCh38.p14)	T	0.099	*LOC105374773*	1.278 [0.681–2.398]	0.445
	rs77464636	chr7:2301325 (GRCh38.p14)	A	0.103	*SNX8*	0.85 [0.459–1.574]	0.605
	rs7525555	chr1:202170575 (GRCh38.p14)	G	0.138	*PTPRVP*	0.747 [0.420–1.329]	0.321
	rs7175639	chr15:50210373 (GRCh38.p14)	C	0.206	*SLC27A2*	1.005 [0.609–1.660]	0.983
	rs117920703	chr9:34250372 (GRCh38.p14)	A	0.060	*UBAP1*	0.449 [0.185–1.093]	0.078
	rs28438600	chr8:15523840 (GRCh38.p14)	A	0.050		0.598 [0.252–1.418]	0.243
	rs17134252	chr11:99717399 (GRCh38.p14)	A	0.059	*CNTN5*	0.978 [0.448–2.133]	0.955
	**rs55870181**	chr14:84615718 (GRCh38.p14)	T	0.272		1.821 [1.123–2.951]	**0.015**
	rs147699000	chr22:44353381 (GRCh38.p14)	A	0.070		0.901 [0.428–1.899]	0.785
	rs8074518	chr17:14443114 (GRCh38.p14)	G	0.338		0.970 [0.526–1.792]	0.924
	rs73497629	chr9:100688200 (GRCh38.p14)	C	0.242		1.258 [0.768–2.060]	0.362
	rs12429312	chr13:22915866 (GRCh38.p14)	A	0.212	*LINC00621*	0.908 [0.539–1.530]	0.717
	rs149903005	chr13:66587358–64 (GRCh38.p14)	C	0.126	*PCDH9, LOC105370247*	1.133 [0.639–2.011]	0.669
HMDB0000448 Adipic acid	rs6057003	chr20:9908557 (GRCh38.p14)	C	0.179		0.853 [0.520–1.400]	0.529
	rs10846689	chr12:124601819 (GRCh38.p14)	T	0.198		1.056 [0.610–1.826]	0.847
	rs73608605	chr8:39240193 (GRCh38.p14)	G	0.086	*ADAM32*	1.357 [0.730–2.521]	0.334
	rs12361624	chr11:30658520 (GRCh38.p14)	G	0.153		1.422 [0.859–2.354]	0.171
	rs837465	chr12:124534405 (GRCh38.p14)	A	0.188	*NCOR2*	0.943 [0.539–1.647]	0.835
	rs6964529	chr7:54293427 (GRCh38.p14)	C	0.085		1.064 [0.567–1.993]	0.848
	rs6739384	chr2:56142078 (GRCh38.p14)	A	0.064	*LOC105374690*	0.527 [0.248–1.124]	0.097
	rs9291437	chr4:22163632 (GRCh38.p14)	C	0.426		1.163 [0.710–1.907]	0.548
HMDB0013205 9-Decenoylcarnitine	rs117445640	chr4:179766591 (GRCh38.p14)	T	0.116		0.963 [0.556–1.669]	0.894
	rs17116178	chr11:113956604 (GRCh38.p14)	T	0.107		0.787 [0.442–1.399]	0.414
	rs2836817	chr21:39001801 (GRCh38.p14)	C	0.193	*LINC02940*	0.639 [0.390–1.049]	0.077
	rs71364155	chr17:12218522 (GRCh38.p14)	T	0.380		1.101 [0.688–1.762]	0.688
	rs1532216	chr12:99210187 (GRCh38.p14)	A	0.063	*ANKS1B*	1.186 [0.605–2.325]	0.619
HMDB0003759 5a-Pregnane-3,20-dione	rs74869776	chr12:30963077 (GRCh38.p14)	G	0.050	*TSPAN11*	0.716 [0.319–1.607]	0.418
	rs79255083	chr4:66526962 (GRCh38.p14)	C	0.069		1.104 [0.561–2.173]	0.775
	rs7760758	chr6:24036861 (GRCh38.p14)	A	0.062		0.636 [0.295–1.369]	0.247
	rs9792660	chr9:29371008 (GRCh38.p14)	T	0.205		0.802 [0.497–1.295]	0.367

Genome-wide association analysis of significant pancreatic cancer-related metabolites from XGBoost was performed. Significant associations (*p* ≤ 5 × 10^–6^) were presented. Exp(B) and *p* were derived from logistic regression of pancreatic cancer with adjusting sex. Values reported in bold are statistically significant in logistic regression evaluating association between pancreatic cancer and EA of SNP (*p* < 0.05)

*EA*: effect allele, *EAF* effect allele frequency

### Network analysis between metabolomic biomarkers and clinical/biochemical indicators

**Fig. 1:**
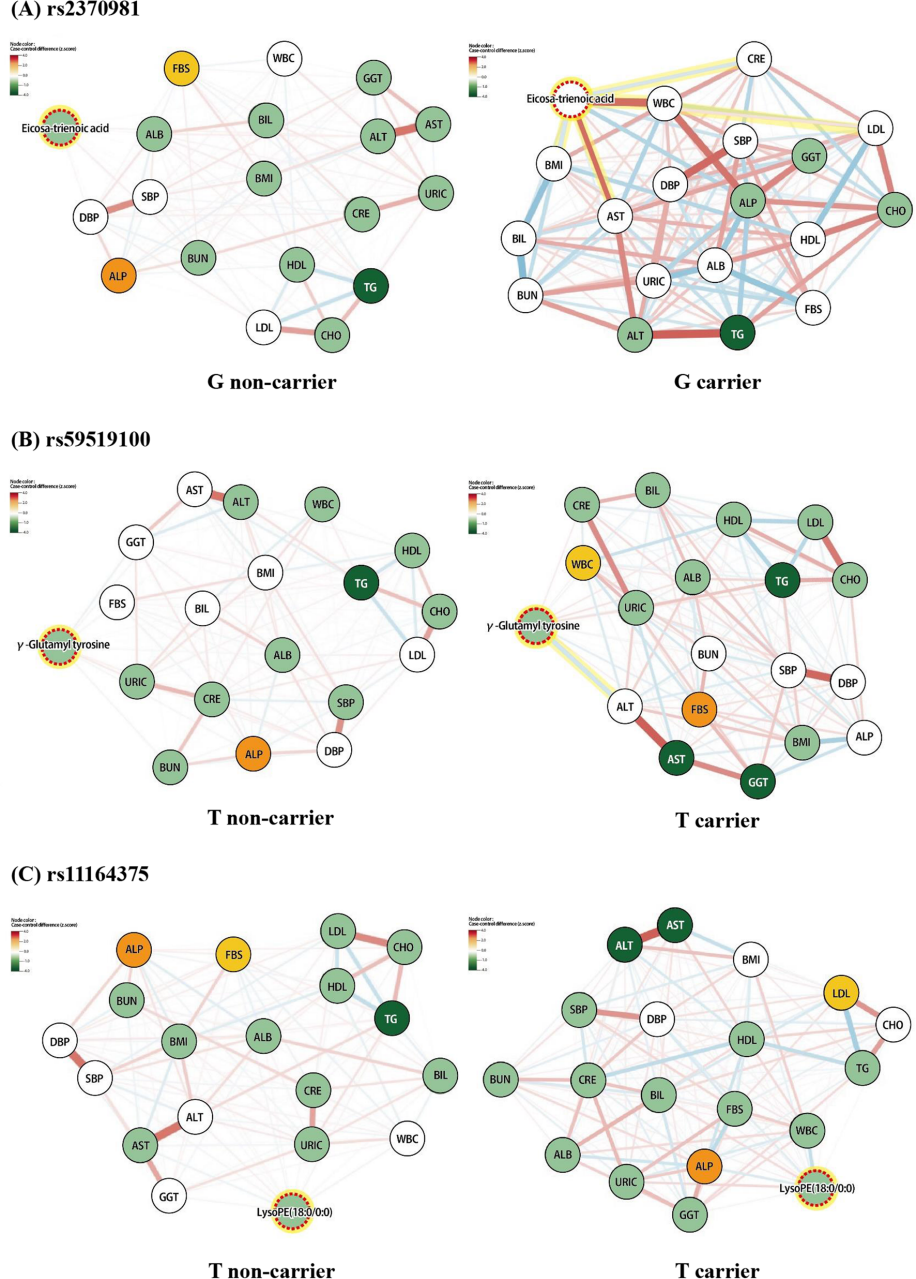

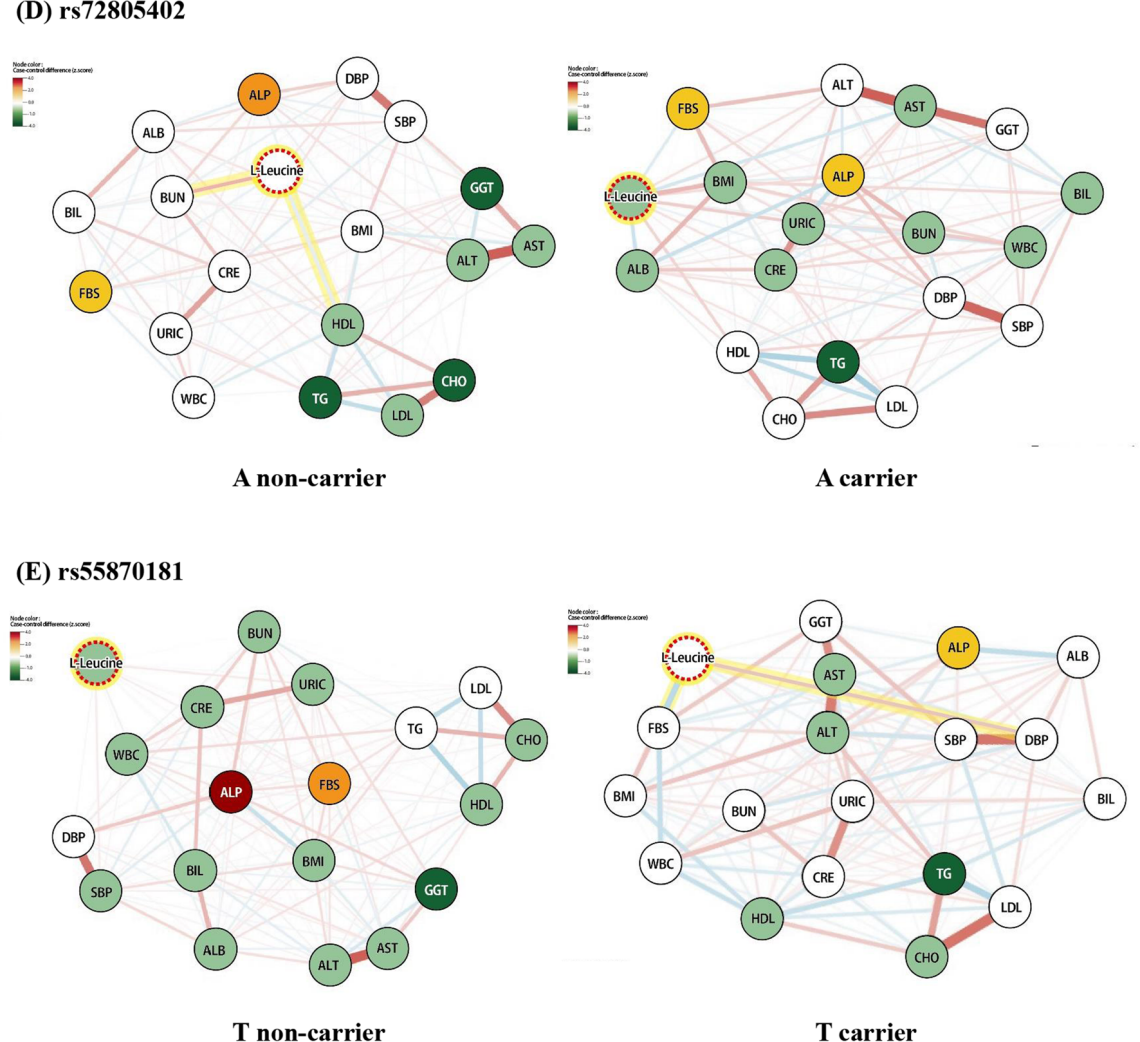
The network between metabolites and clinical/biochemical indicators in each SNP group. *ALB* albumin, *ALP* alkaline phosphatase, *ALT* alanine aminotransferase, *AST* aspartate aminotransferase, *BIL* bilirubin, *BMI* body mass index, *BUN* blood urea nitrogen, *CHO* total cholesterol, *CRE* creatinine, *DBP* diastolic blood pressure, *FBS* fasting blood sugar, *GGT* gamma-glutamyltransferase, *HDL*, high-density lipoprotein, *LDL* low-density lipoprotein, *SBP* Systolic blood pressure, *TG* Triglyceride, *URIC* uric acid, *WBC* white blood cell. Node presents metabolite or clinical/biochemical indicators; the edge between two nodes indicates a partial correlation. The color of the nodes represents the z-score when comparing the pancreatic cancer incidence and control groups. Positive and negative correlations are represented using light-red and light-blue edges. Thicker edges represent stronger correlations between the two metabolite levels

We divided the subjects into each SNP’s effect allele carrier and non-carrier groups. Then, clinical/biochemical indicators and pair metabolites of the SNP were used to create network models based on the z-score obtained after comparing the pancreatic cancer incidence and control groups for each variable and the partial correlation values between them (Fig. 1).

As a result, pair metabolites of rs2370981, rs55870181, rs59519100, and rs72805402 displayed significantly different partial correlation network patterns with the clinical/biochemical indicators on comparison of the effect allele carrier and the non-carrier groups of each SNP. In summary, the risk allele carriers of rs2370981 showed several significant partial correlations that were not detected in the non-risk allele carriers; eicosa-11,14,17-trienoic acid with low-density lipoprotein (LDL) (*r* = 0.613, *p* = 0.045), alanine aminotransferase (ALT) (*r* = 0.632, *p* = 0.037), white blood cell (*r* = 0.816, *p* = 0.002), body mass index (*r* = -0.636, *p* = 0.036), and creatinine (*r* = − 0.67, *p* = 0.024). Moreover, a significant negative partial correlation between γ-glutamyl tyrosine and aspartate aminotransferase (AST) (*r* = − 0.237, *p* = 0.049) was observed in the risk allele carriers of rs59519100. Finally, l-leucine exhibited notable partial correlations with a few clinical/biochemical indications. l-Leucine and diastolic blood pressure (*r* = 0.18, *p* = 0.046) and L-leucine and glucose (*r* = − 0.259, *p* = 0.004) were identified as the risk allele carriers of rs55870181. In addition, in the non-risk allele carriers of rs72805402, l-leucine positively correlated with the blood urea nitrogen level (*r* = 0.137, *p* = 0.049) and negatively correlated with high-density lipoprotein (*r* = − 0.146, *p* = 0.035).

### Mediation and moderation analyses

**Fig. 2:**
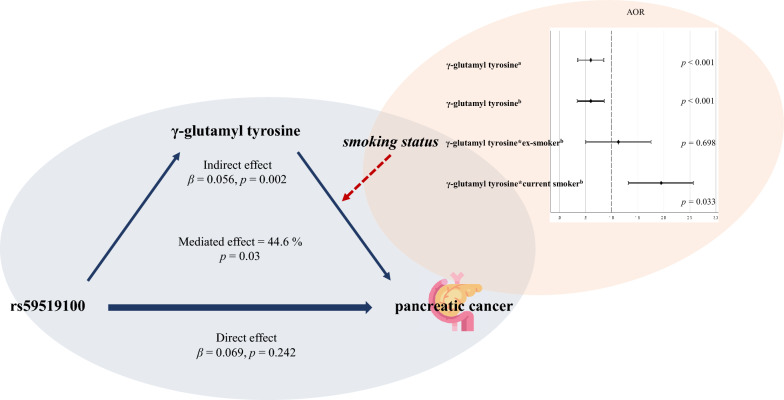
Mediation and moderation analysis. The result of the mediation analysis is presented in the blue circle and that of the moderation analysis in the red circle. Adjusting odds ratio (AOR) and confidence interval are indicated with points and lines on the graph. Variables marked with **a** are derived from the age- and sex-adjusting model. Variable marked with **b** is derived from the age-, sex-, and smoking status-adjusting model

Mediation analysis, after adjusting for age and sex, was conducted on the selected metabolites and SNP biomarkers for pancreatic cancer. We noted significant outcomes in the association between γ-glutamyl tyrosine and rs59519100. Although rs59519100 showed no significant direct effect on pancreatic cancer incidence (*β* = 0.069, *p* = 0.242), γ-glutamyl tyrosine mediated the indirect effect of rs59519100 on pancreatic cancer incidence (*β* = 0.056, *p* = 0.002) with causal mediation effects of 44.6% relative to the total effect (Fig. 2).

Next, we conducted a moderation analysis after adjusting for the age and sex so as to explore the effect of smoking status as a moderator on the association among γ-glutamyl tyrosine, rs59519100, and pancreatic cancer (Fig. 2). The level of γ-glutamyl tyrosine was negatively associated with pancreatic cancer risk (*β* = -0.504, *p* < 0.001). It was maintained after adjusting the smoking status (*β* = − 0.508, *p* < 0.001). When the interaction effect (smoking status * γ-glutamyl tyrosine) was added to the linear model, this interaction term was found to be positively associated with pancreatic cancer risk (*β* = 0.666, *p* = 0.033). In other words, the smoking status affected the association between γ-glutamyl tyrosine and pancreatic cancer risk. In addition, smoking did not significantly modulate the other associations (Additional file 1: Figure S3).

### Evaluation of the predictive power as a biomarker for pancreatic cancer

**Fig. 3:**
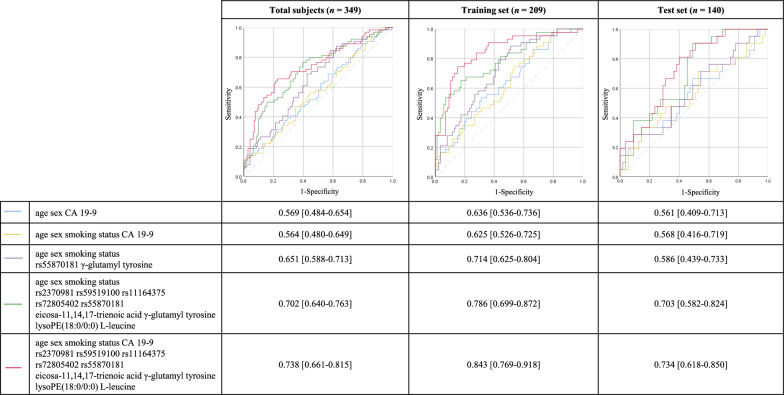
ROC curves for the prediction of pancreatic cancer in total subjects. Prediction models in the total subjects (*n* = 349), training set (*n* = 209), and test set (*n* = 140). The variables utilized in each model are different, and each model is displayed in a different color

Figure 3 depicts the prediction model using conventional risk factors and significant biomarkers identified in the present research. First, the total subjects' results (*n* = 349) are as follows: an area under the curve (AUC) obtained from the prediction model consisting of age, sex, and CA 19–9 was 0.569 [0.484–0.654]. The conventional model with age, sex, smoking status (never, ever, current), and CA 19–9 was 0.564 [0.480–0.649]. On adding five SNP biomarkers (i.e., rs2370981, rs59519100, rs11164375, rs72805402, and rs55870181) and four metabolic biomarkers (i.e., eicosa-11,14,17-trienoic acid, γ-glutamyl tyrosine, lysoPE(18:0/0:0), and L-leucine) to the conventional model, AUC was improved to 0.702 [0.640–0.763]. The highest AUC of 0.738 [0.661–0.815] was observed in the final model consisting of all variables (i.e., age, sex, smoking status, CA 19–9, rs2370981, rs59519100, rs11164375, rs72805402, rs55870181, eicosa-11,14,17-trienoic acid, γ-glutamyl tyrosine, lysoPE(18:0/0:0), and l-leucine). Furthermore, the predictive power of the model using variables indicating significance in mediation and moderation analyses (i.e., age, sex, smoking status, γ-glutamyl tyrosine, and rs59519100) was an AUC of 0.651 [0.588–0.713], which was within the range of predictive power of the previously described models.

The prediction performance trend was similar even when analyzed separately into training (*n* = 209) and test sets (*n* = 140). In both sets, the final model when metabolic and SNP biomarkers were added to the conventional model exhibited the most potent prediction power, and the predictive power of the final model was considerably improved when compared to the conventional model. The final model of the training set had an AUC of 0.843 [0.769–0.918], whereas the conventional model was 0.625 [0.526–0.725]. In addition, the final model of the test set had an AUC of 0.734 [0.618–0.850], while the conventional model showed 0.568 [0.416–0.719].

## Discussion

We discovered four metabolites (i.e., eicosa-11,14,17-trienoic acid, γ-glutamyl tyrosine, lysoPE(18:0), and L-leucine) and five SNPs (i.e., rs2370981, rs59519100, rs11164375, rs72805402, and rs55870181) with the potential to act as predictive biomarkers for pancreatic cancer using metabolite-GWAS analysis. As the current study used data from subjects obtained before the onset of pancreatic cancer, no significant difference was noted between the two groups in terms of CA 19–9, which was mainly used to determine the prognosis, treatment effects, and recurrence of pancreatic cancer. Moreover, the predictive value of the conventional model for predicting pancreatic cancer using age, gender, smoking status, and CA 19–9 was 0.564 [0.480–0.649]. However, when the four metabolites and five SNPs identified in this study were combined, the predictive power for pancreatic cancer increased to 0.702 [0.640–0.763], and, when CA 19–9 was integrated, the predictive power for pancreatic cancer was found to be the highest, with an AUC of 0.738 [0.661–0.815]. In other words, utilizing CA 19–9, not extensively used in the screening tests owing to its low specificity, with the biomarkers revealed in our study could improve the predictive potential for the early detection of pancreatic cancer risk. Furthermore, the partial correlation network between each pair of metabolites and clinical/biochemical indicators revealed significantly different patterns according to the effect allele carrier or non-carrier groups of rs2370981, rs55870181, rs59519100, and rs72805402; metabolism involving metabolic biomarkers were associated with a genetic predisposition.

Among them, the indirect effect of rs59519100 mediated by γ-glutamyl tyrosine on pancreatic cancer risk was demonstrated through mediation analysis. Furthermore, the association between γ-glutamyl tyrosine and pancreatic cancer risk was impacted by the smoking status. γ-Glutamyl tyrosine is a dipeptide composed of γ-glutamate and tyrosine—a product of incomplete proteolytic breakdown. Although dipeptides have some physiological effects, the metabolic function of γ-glutamyl tyrosine is unclear. We observed a higher serum level of γ-glutamyl tyrosine in the pancreatic cancer incidence group. The abnormal levels of γ-glutamyl dipeptide have been linked to several metabolic disorders in epidemiological studies [13, 14]. Similarly, metabolomics discovered several γ-glutamyl dipeptides related to oxidative stress and dysregulated lipid profiles [15, 16] as they are involved in the γ-glutamyl cycle for regenerating the intracellular glutathione. As γ-glutamyltransferase (GGT) detoxicates glutathione, increased GGT activity is an important marker for increased oxidative stress. γ-Glutamyl tyrosine, observed in our study, may also contribute to the biochemical pathways, inducing oxidative stress.

Unexpectedly, γ-glutamyl tyrosine was not significantly correlated with the levels of GGT, ALT, and AST in all the subjects of the present study (data not shown). However, a negative partial correlation between γ-glutamyl tyrosine and AST (*r* = − 0.237, *p* = 0.049) was identified in the risk allele carrier of the rs59519100 group. In other words, subjects with the rs59519100 risk allele showed a high risk of developing pancreatic cancer, and metabolic alterations in their etiology were implied by AST and γ-glutamyl tyrosine. As liver enzymes (i.e., GGT, ALT, and AST) are very close to each other, the significance of AST could be connected with the mechanisms of γ-glutamyl tyrosine linked to GGT. Indeed, pancreatic ductal adenocarcinoma patients with elevated AST levels revealed a considerably shorter overall survival than those with lower AST levels [17]. Furthermore, we discovered a novel SNP, rs59519100, significantly associated with γ-glutamyl tyrosine, in relation to the risk of pancreatic cancer. Further study is therefore needed to clarify the underlying mechanisms of these valuable biomarkers.

Intriguingly, through moderation analysis, we demonstrated that the smoking status significantly affected the association between γ-glutamyl tyrosine and pancreatic cancer risk. On the other hand, an association between the smoking status and γ-glutamyl tyrosine has not yet been reported, while liver enzymes (such as GGT, AST, and ALT), which is possibly connected to γ-glutamyl tyrosine, has shown some evidence of association with the smoking habit. Zhang et al*.* [18] determined the smoking and alcohol drinking habit synergistically affected the elevation of GGT levels in Chinese [19, 20]. In a mouse model, the maternal smoking exposure during pregnancy increased the severity of non-alcoholic steatohepatitis in offspring mice by increasing their serum ALT, AST, total cholesterol, and triglyceride levels and modulating the phosphorylation of AMP-activated protein kinase [21]. Elucidation of the exact metabolic pathways between these biomarkers through which the smoking modulates can facilitate precision medicine or management for pancreatic cancer.

The next notable biomarker is l-leucine, which belongs to the branched-chain amino acids (BCAAs). The breakdown of BCAAs, mainly stored as tissue protein, provides a source for synthesizing other molecules. Consistent with some previous reports, serum l-leucine was elevated in the prediagnostic serum of the pancreatic cancer-incidence group when compared to the control in our research. Mayers et al. observed that subjects with elevated circulating BCAAs in the prediagnostic plasma had more than a two-fold increased risk of pancreatic ductal adenocarcinoma (PDAC) [22]. The leading cause of this increase in plasma BCAAs is tissue protein degradation exceeding the systemic requirement for BCAAs [22, 23], which often occurs in metabolic diseases [24]. Moreover, abnormal physiological functions of the pancreas, including that related to insulin secretion, could directly modulate tissue protein degradation, including that of BCAAs. In all the study subjects, l-leucine was found to be negatively correlated with the levels of glucose (*r* = − 0.113, *p* = 0.034), LDL (*r* = − 0.130, *p* = 0.015), and uric acid (*r* = − 0.118, *p* = 0.031) (data not shown). These findings indicate that higher leucine levels in the pancreatic cancer incidence group may closely reflect the condition of the pancreas during disease progression.

Furthermore, one of the two SNPs associated with L-leucine was mapped to the gene; rs72805402 mapped to *ZNF503* (Zinc Finger Protein 503) that functions as a transcriptional repressor. Rich leucine residues in the SCAN domain of zinc finger proteins participate in protein—protein interaction, thereby inducing various transcription activities [25]. The associations of *ZNF503* acting as an essential regulator have been reported during the developmental process and tumor initiation with multiple carcinomas, [26, 27] but not in pancreatic cancer. Therefore, our data provide a candidate gene for diagnostic and therapeutic strategies for pancreatic cancer. Different network patterns in the risk allele carrier or non-carrier groups provide a comprehensive insight into SNP-metabolite-clinical indicators of pancreatic cancer incidence.

Finally, eicosa-11,14,17-trienoic acid associated with rs2370981 mapped to *NRXN3* (neurexin 3) belongs to the long-chain fatty acids, with very few articles published on eicosa-11,14,17-trienoic acid [28]. *NRXN3* encodes the receptor and cell adhesion molecules mainly involved in the nervous system [29]. Therefore, most mutations in this gene have been reported in neurological diseases, and several associations with carcinoma have been reported, albeit not in pancreatic cancer. Interestingly, hypermethylation of ZNF582, the same class as zinc finger protein associated with L-leucine in our research, regulated the transcription of *NRXN3* in nasopharyngeal carcinoma [30]. In addition, the changes in the protein NRXN3 level in the brain cerebrospinal fluid derived from Huntington’s disease agreed with the protein and mRNA levels of ZNF503 [31]. Based on the recent literature review, we suggested that SNPs of the two genes discovered in our study could synergistically affect the pancreatic cancer risk.

Several limitations should be delineated in this case. First, this study was conducted on design without classifying the pancreatic cancer type. Therefore, if the result was replicated from blood samples collected following the pancreatic cancer stage with type information, the biomarkers identified in the present study could be robust for pancreatic cancer. Next, it was a small sample size for conducting GWAS. With a larger sample size, it was possible to discover more meaningful biomarkers, with more substantial statistical power. Third, drawing the causality and interpreting the underlying mechanisms between biomarkers were challenging in our study design. Instead, we performed moderation, mediation, and network analysis. Additional experimental research is therefore warranted to elucidate the exact mechanism of pathogenesis related to discovered associations. Furthermore, the effect of smoking was analyzed using only self-reported smoking status data. Thus, it is necessary to examine the impact of smoking on other variables, such as the duration and amount of tobacco use.

Despite some limitations in this study, it is the first one to employ metabolite-GWAS for pancreatic cancer in the Korean population. As a result, we identified four metabolites (i.e., eicosa-11,14,17-trienoic acid, γ-glutamyl tyrosine, lysoPE(18:0), and L-leucine) and five SNPs (i.e., rs2370981, rs59519100, rs11164375, rs72805402, and rs55870181) with the potential for use as predictive biomarkers for pancreatic cancer risk. Particularly, we noted the indirect effect of rs59519100 mediated by γ-glutamyl tyrosine on pancreatic cancer risk and affected by the smoking status. Indeed, the smoking status affected the newly discovered pathogenesis involving γ-glutamyl tyrosine related to pancreatic cancer risk. In addition, the difference in the network pattern based on the presence or absence of risk allele of SNP is also noteworthy. We therefore believe that the present results can serve as the base of precision medicine or management for pancreatic cancer.

### Supplementary Information

**Additional file 1: Figure S1.** Heatmap of metabolite abundance in each group. **Figure S2.** Manhattan plot from GWAS. **Figure S3.** Moderation effect of smoking on association between metabolite and pancreatic cancer risk.

**Additional file 2: Data S1.** Python and R codes used in the current research. **Data S2. **Characteristics of the divided set from XGBoost.

## Data Availability

Some or all datasets generated during and/or analyzed during the current study are not publicly available, but can be made available from the corresponding author upon reasonable request.

## Abbreviations

ALT: Alanine aminotransferase
AST: Aspartate aminotransferase
AUC: Area under the curve
BCAAs: Branched-chain amino acids
CA: Carbohydrate antigen
ESI: Electrospray ionization mode
GGT: Gamma-glutamyltransferase
ISTD: Internal standard
KCPS: Korean Cancer Prevention Study
KEGG: Kyoto encyclopedia of genes and genomes
LDL: Low-density lipoprotein
NRXN3: Neurexin 3
PDAC: Pancreatic ductal adenocarcinoma
QC: Quality control
SNP: Single nucleotide polymorphisms
XGBoost: EXtreme Gradient Boosting
ZNF503: Zinc finger protein 503
