# Supplementary material for: Non-invasive biomarkers for early diagnosis of pancreatic cancer risk: metabolite genomewide association study based on the KCPS-II cohort
Source: J Transl Med. 2023 Dec 4;21:878. doi: 10.1186/s12967-023-04670-x (PMC10694897; doi:10.1186/s12967-023-04670-x)

Supplementary figure 1. Heatmap of metabolite abundance in each group

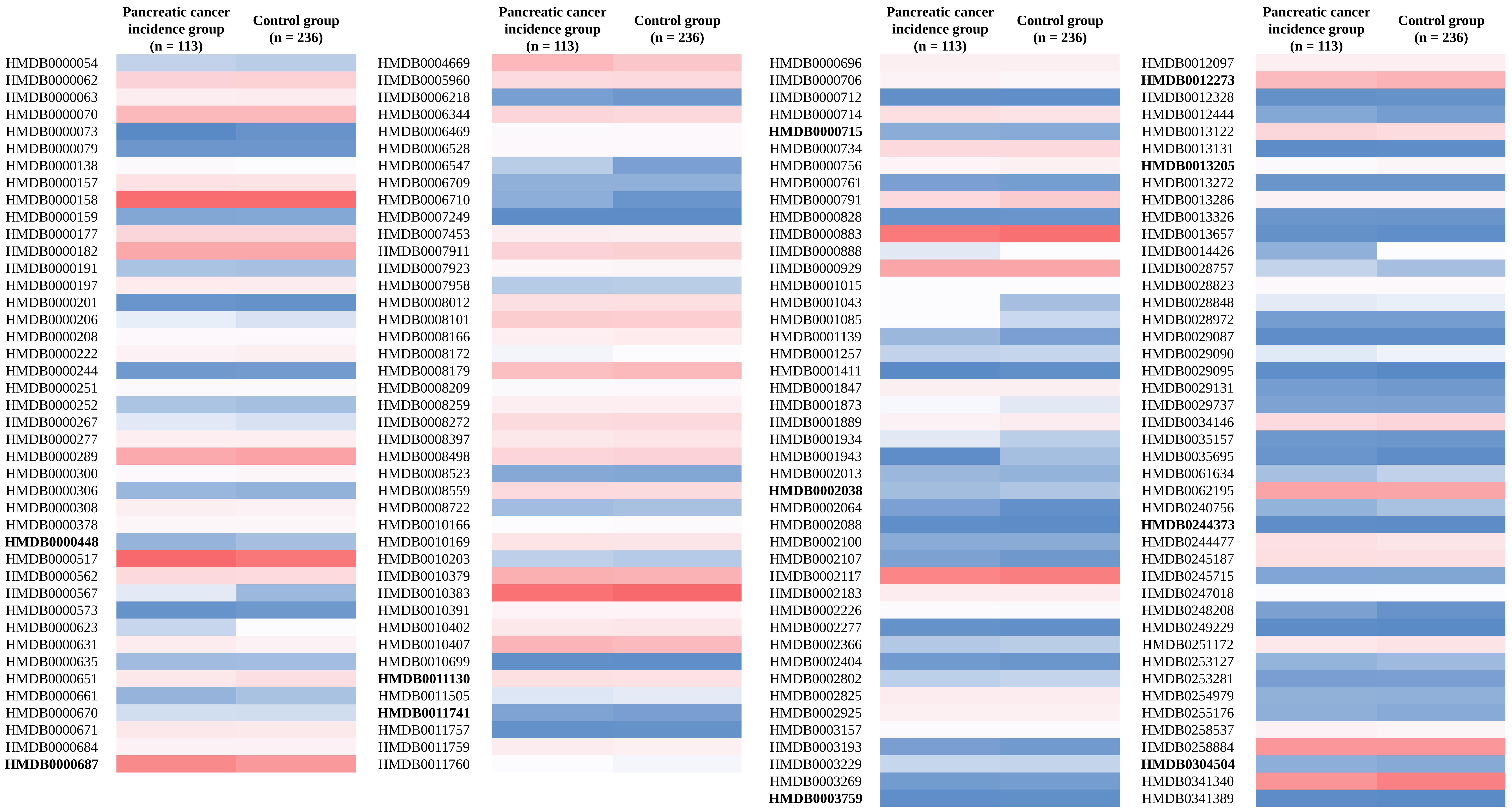

*Significant Metabolites in OPLS-DA (VIP>1.0) are written in **bold.**

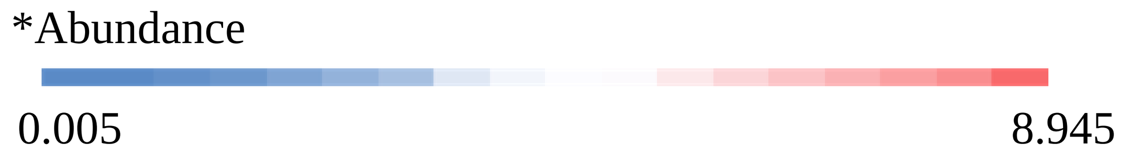

Supplementary figure 2. Manhattan plot from GWAS

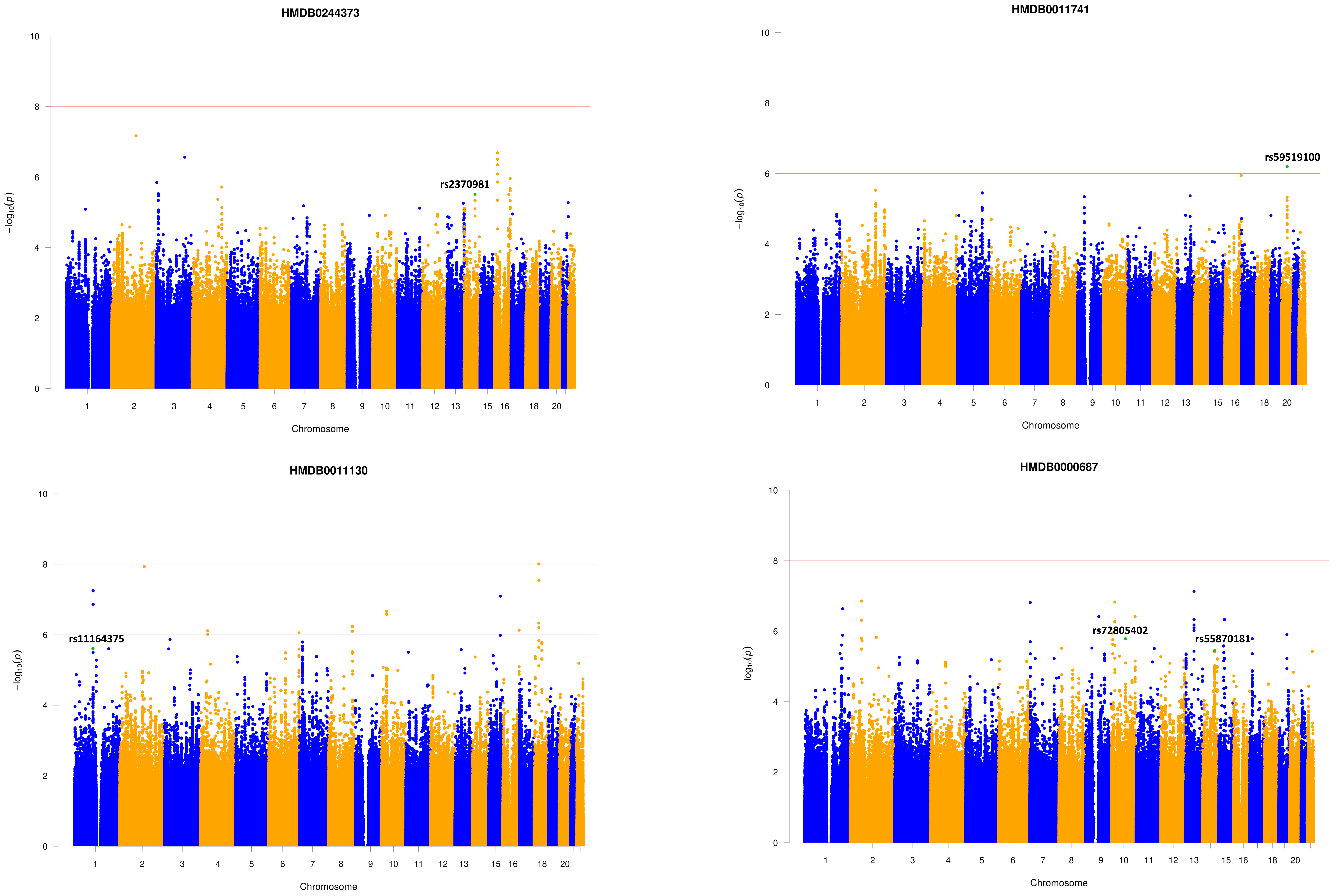

Supplementary figure 3. Moderation effect of smoking on association between metabolites and pancreatic cancer risk

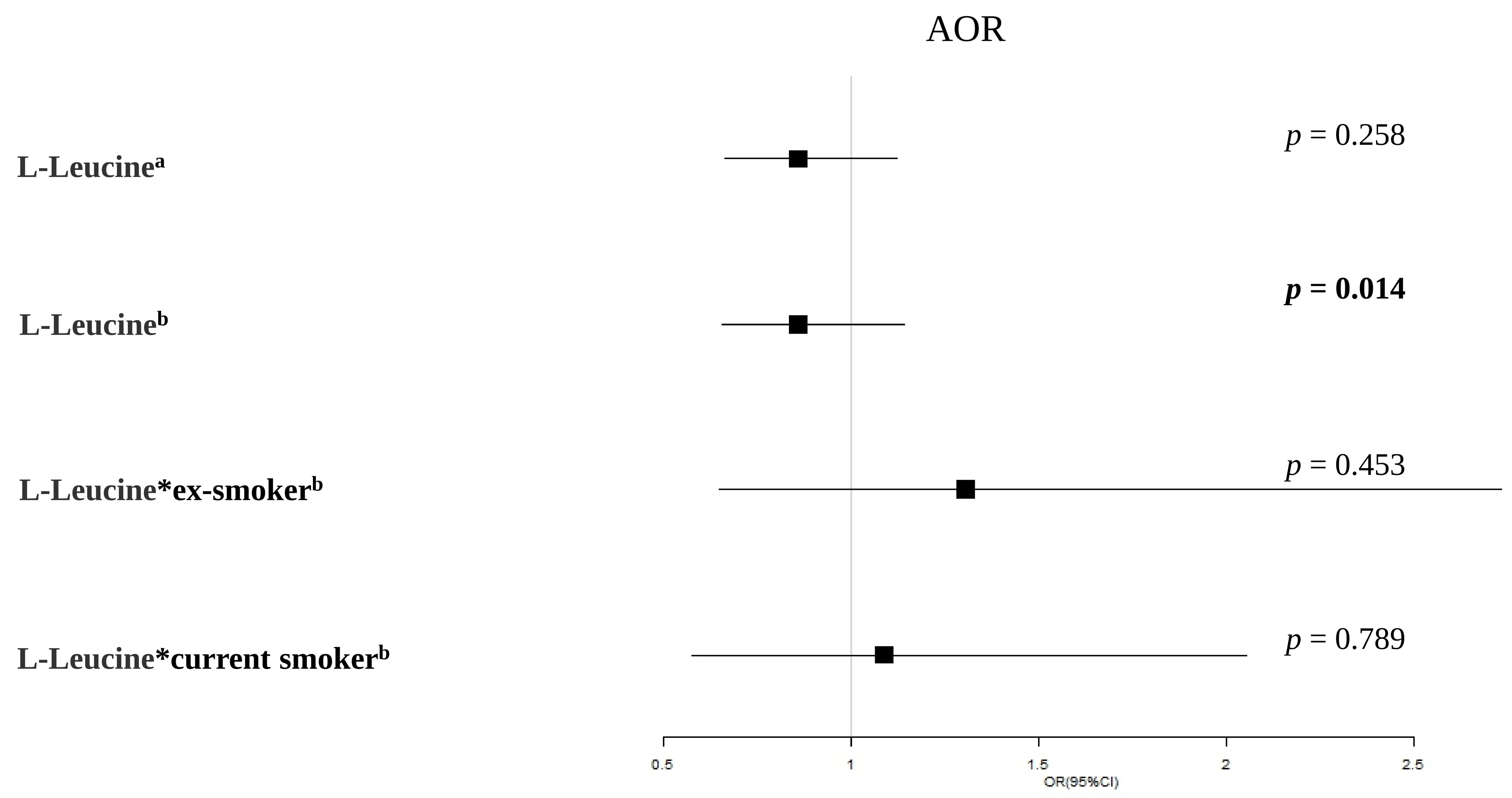

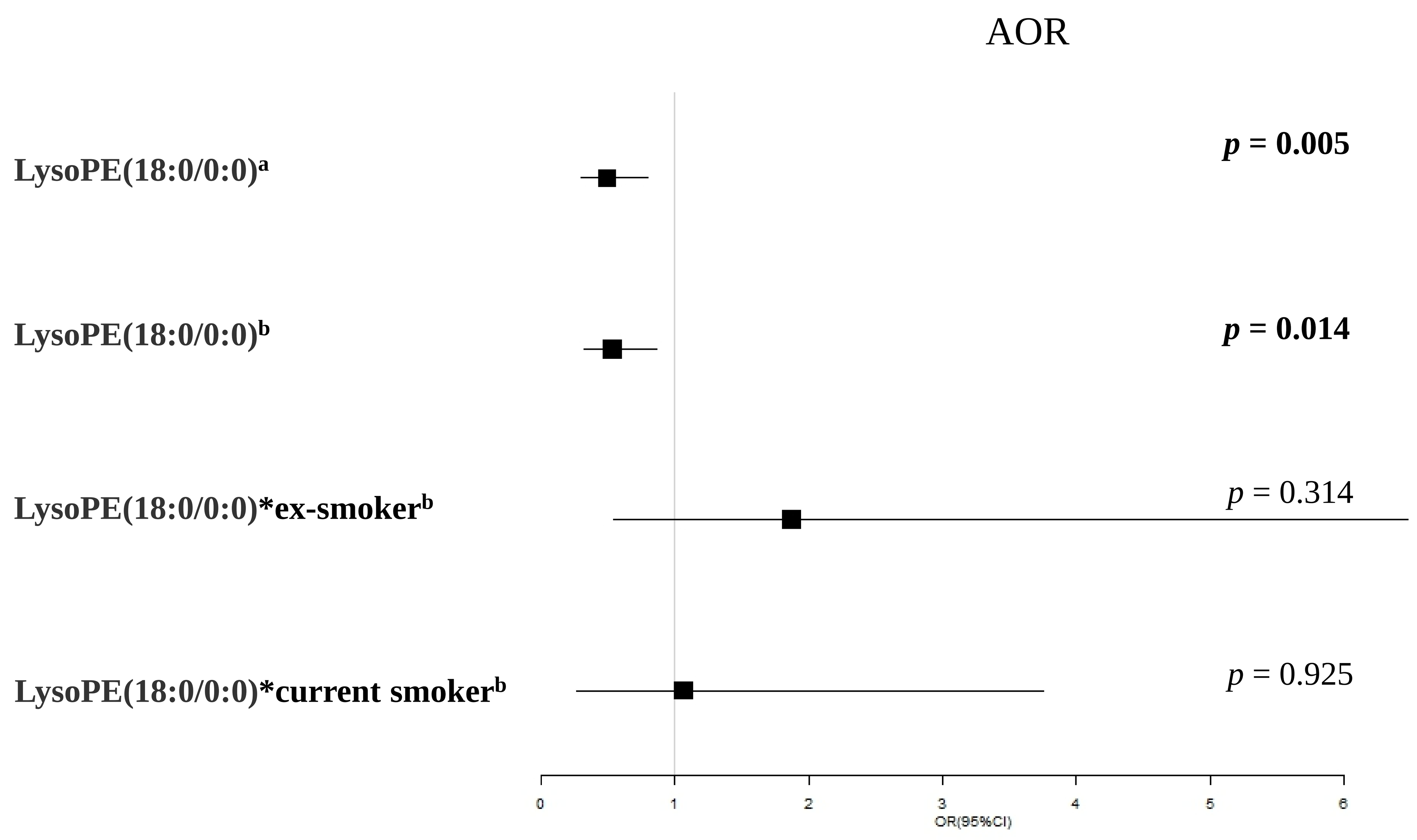

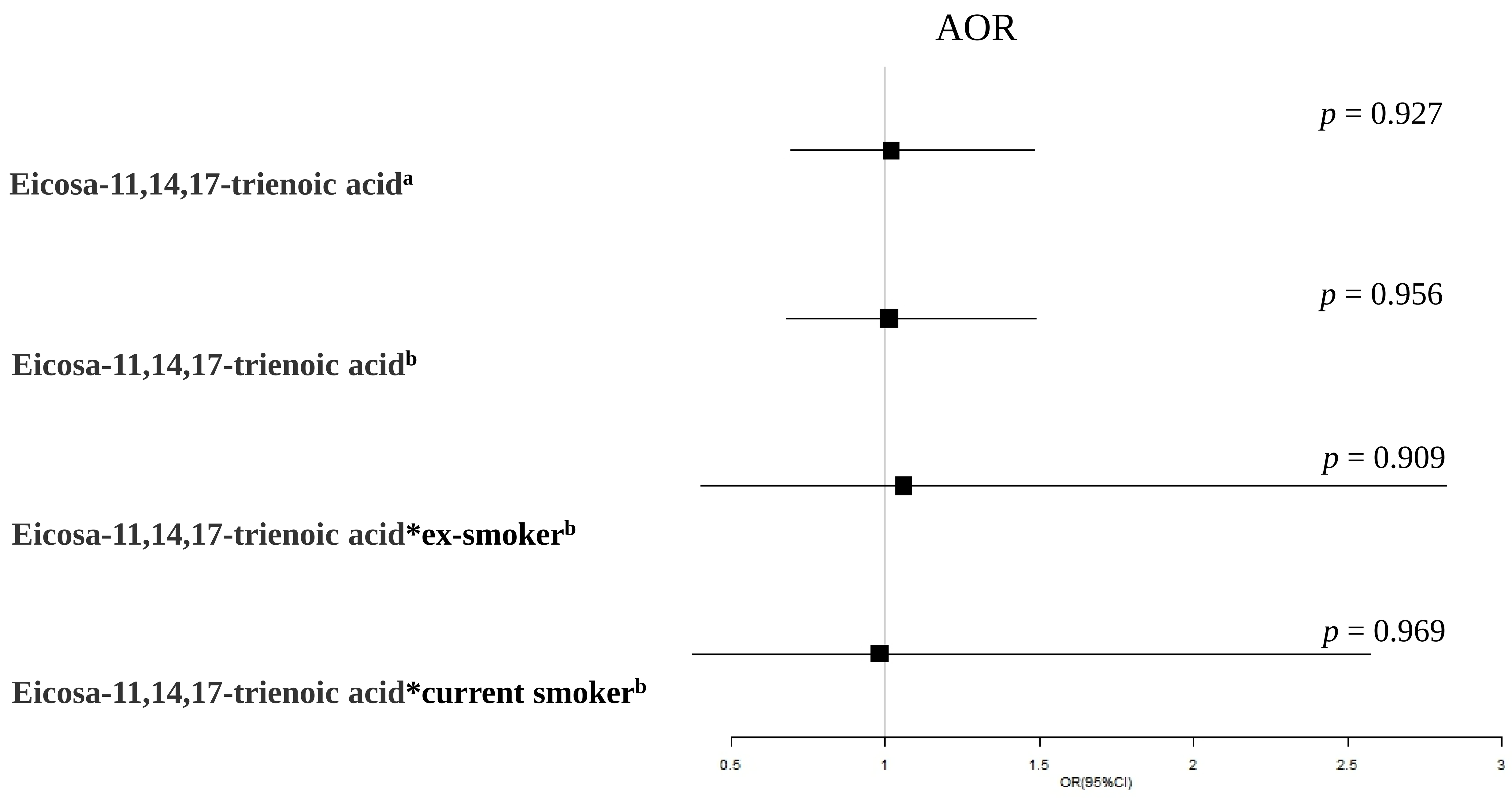

Supplement: Supplementary file 1 — Additional file 1: Figure S1. Heatmap of metabolite abundance in each group. Figure S2. Manhattan plot from GWAS. Figure S3. Moderation effect of smoking on association between metabolite and pancreatic cancer risk. [file 12967_2023_4670_MOESM1_ESM.pdf]
