# Supplementary material for: Non-invasive biomarkers for early diagnosis of pancreatic cancer risk: metabolite genomewide association study based on the KCPS-II cohort
Source: J Transl Med. 2023 Dec 4;21:878. doi: 10.1186/s12967-023-04670-x (PMC10694897; doi:10.1186/s12967-023-04670-x)
Supplement: Supplementary file 2 — Additional file 2: Data S1. Python and R codes used in the current research. Data S2. Characteristics of the divided set from XGBoost. [file 12967_2023_4670_MOESM2_ESM.pdf]

Supplementary data 1. Python and R codes used in the current research

***XGBoost**

```
# import library
import requests as rq
import pandas as pd
import urllib.request
import numpy as np
import matplotlib.pyplot as plt
import sklearn
import graphviz
import xgboost
import seaborn as sns
from sklearn.feature_selection import SelectKBest
from sklearn.feature_selection import chi2
from xgboost import XGBClassifier
from sklearn.datasets import load_breast_cancer
from sklearn.model_selection import train_test_split, GridSearchCV
from sklearn.metrics import accuracy_score
from xgboost import XGBClassifier
from sklearn.preprocessing import LabelEncoder
from sklearn.metrics import confusion_matrix, accuracy_score
from sklearn.metrics import precision_score, recall_score
from sklearn.metrics import f1_score, roc_auc_score

# import data
data =pd.read_excel("file name.xlsx")

# data split
data_x = data.iloc[:,2:]
data_y = data["Label"]
np.random.seed(8)
from sklearn.model_selection import train_test_split
x_train, x_test, y_train, y_test = train_test_split(data_x, data_y, test_size=0.4, random_state=121)
le = LabelEncoder()
```

```
y_train = le.fit_transform(y_train)
from xgboost import XGBClassifier
evals = [(x_test, y_test)]

xgb_wrapper = XGBClassifier(eta = 0.1,
                            n_estimators=50,
                            learning_rate=0.15,
                            max_depth=2,
                            min_child_weight = 5,
                            alpha=0.001)
xgb_wrapper.fit(x_train, y_train, early_stopping_rounds=200,eval_set=evals, eval_metric="logloss",    verbose=True)

y_preds = xgb_wrapper.predict(x_test)
y_pred_proba = xgb_wrapper.predict_proba(x_test)[:, 1]

# function for performance
def get_clf_eval(y_test, pred=None, pred_proba=None):
    confusion = confusion_matrix( y_test, pred)
    accuracy = accuracy_score(y_test , pred)
    precision = precision_score(y_test , pred )
    recall = recall_score(y_test , pred)
    f1 = f1_score(y_test,pred)
    # add ROC-AUC
    roc_auc = roc_auc_score(y_test, pred_proba)
    print(confusion)
    # ROC-AUC print
    print('accuracy: {0:.4f}, precision : {1:.4f}, recall : {2:.4f},\
    F1: {3:.4f}, AUC:{4:.4f}'.format(accuracy, precision, recall, f1, roc_auc))

# get performance
get_clf_eval(y_test, y_preds, y_pred_proba)
```

***Mediation analysis**

```
library(mediation)
df_M <- df[df$SEX==1,]
```

```
df_F <- df[df$SEX==2,]
df$SMOK_fac2 <- ifelse(df$SMOK_fac=="1", 0, 1)

#Association between X-Y
XY_M1 <- glm(Label ~ SMOK_fac1+AGE+SEX, family = binomial(), data = df)
XY_F1 <- glm(Label ~ SMOK_fac+AGE, family = binomial(), data = df_F)
XY_F2 <- glm(Label ~ SMOK_fac2+AGE, family = binomial(), data = df_F)
summary(XY_M1)
summary(XY_F2)

#/******** HMDB0011741 ********/
HMDB0011741_C <- lm(HMDB0011741 ~ SMOK_fac2+AGE, data = df_F)
Y_HMDB0011741_C <- glm(Label ~ HMDB0011741 + SMOK_fac2+AGE, family = binomial(), data = df_F)
resHMDB0011741_C <- mediate(HMDB0011741_C,Y_HMDB0011741_C,
                            treat="SMOK_fac2", mediator = "HMDB0011741",
                            boot=T, sims=999)
summary(resHMDB0011741_C)
```

***Moderation analysis**

```
setwd("working directory")
moderation <- read.csv("newdf.csv")

#/******** HMDB0011741 ********/
HMDB0011741_1 <- glm(Label~HMDB0011741 +AGE+as.factor(SEX), data=moderation, family=binomial(link = "logit"))
HMDB0011741_2 <- glm(Label~HMDB0011741 +AGE+as.factor(SEX)+ as.factor(SMOK), data=moderation, family=binomial(link = "logit"))
HMDB0011741_3 <- glm(Label~HMDB0011741*as.factor(SMOK)+AGE+as.factor(SEX), data=moderation, family=binomial(link = "logit"))

# PANCR ~ metabololites+AGE+SEX
summary(HMDB0011741_1) ; confint(HMDB0011741_1)
# PANCR ~ metabololites+AGE+SEX+SMOK
summary(HMDB0011741_2) ; confint(HMDB0011741_2)
# PANCR ~ metabololites+AGE+SEX+metabololites*SMOK
summary(HMDB0011741_3) ; confint(HMDB0011741_3)
```

Supplementary data 2. Characteristics of the divided set from XGBoost

	Training set (*n*=209)			Test set (*n*=140)		
	**Control (*n*=141)**	**Pancreatic cancer incidence (*n*=68)**	***p***	**Control (*n*=95)**	**Pancreatic cancer incidence (*n*=45)**	***p***
Baseline characteristic						
**Age (year)**	52.2±0.767	52.9±1.064	0.608	52.6±0.920	52.1±1.35	0.565
**Male/Female *n*,(%)**	102 (72.3) / 39 (27.7)	57 (83.8) / 11 (16.2)	0.141	72 (75.8) / 23 (24.2)	30 (66.7) / 15 (33.3)	0.257
**Current smoker *n*,(%)**	43 (30.5)	21 (30.9)	**0.018**	27 (28.4)	12 (26.7)	0.924
XGBoost model performance						
**Accuracy**	0.952			0.671		
**Precision**	0.983			0.471		
**Recall**	0.868			0.178		
**AUC**	0.998			0.640		

Mean ± standard error (SE). Comparisons were conducted between the two groups (Control *vs.* Pancreatic cancer incidence) of each set. Age was tested by an independent t-test. Smoking status and sex distribution were tested by a Chi-squared test. Accuracy = (True positive + True negative) / # of total data set, Precision = True positive / (True positive + False positive), Recall = True positive / (True positive + False negative).
